# Associations between oral contraceptive use and risks of hypertension and prehypertension in a cross-sectional study of Korean women

**DOI:** 10.1186/1472-6874-13-39

**Published:** 2013-10-21

**Authors:** Hyejin Park, Kisok Kim

**Affiliations:** 1 Department of International Medical Management, Catholic University of Daegu, Kyungbuk 712-702, Republic of Korea; 2 Department of Pharmacy, Keimyung University, Daegu 704-701, Republic of Korea

**Keywords:** Oral contraceptive, Blood pressure, Hypertension, Prehypertension, KNHANES

## Abstract

**Background:**

The effect of commonly used oral contraceptives (OCs) on blood pressure is still unclear. The aim of this study was to examine the relationship between OCs use and blood pressure and the prevalence of hypertension in a population-based sample of Korean women.

**Methods:**

A cross-sectional study was conducted using data from 3356 participants aged 35–55 years collected in the 2007–2009 Korea National Health and Nutrition Examination Surveys. OC use and demographic characteristics were obtained from participants using a questionnaire, and blood pressure was measured with a mercury sphygmomanometer.

**Results:**

Longer duration of OC use was positively associated with increasing levels of systolic blood pressure and diastolic blood pressure (p for trend <0.001). After adjusting for covariates, the odds ratio (OR) of hypertension was significantly increased in longer-term (>24 months) OC users (OR 1.96; 95% confidence interval (CI) 1.03–3.73) compared with those who had never used OCs. Additionally, use of OCs >24 months was associated with increased odds of prehypertension (adjusted OR 2.23; 95% CI 1.28–3.90) and hypertension or prehypertension (adjusted OR 2.13; 95% CI 1.37–3.32).

**Conclusion:**

This study found a statistically significant association between OC use and blood pressure or hypertension among Korean women.

## Background

Hypertension is one of the most prevalent and etiologically significant risk factors for cardiovascular disease [1]. Although various approaches have been applied in the general population for the prevention, treatment, and control of high blood pressure, hypertension remains a major public health concern [2]. Among women, more than 25% of the world adult population is hypertensive, and an estimated 23.9% of Korean women aged 30 years or older are hypertensive [3,4]. Furthermore, the prevalence of hypertension is predicted to increase more in women than in men [5]. Hypertension contributes to more excess deaths in women than does any other preventable cardiovascular disease risk factor. It is considered a complex-trait disease resulting from interactions between genetic and environmental factors, including oral contraceptive (OC) use [6,7].

OCs are an important and widely accepted contraceptive modality worldwide [8]. In Korea, most OCs contain 20–40 mg ethinyl estradiol as the estrogen component in addition to a progestin component. Although the incidence of OC-induced adverse effects is thought to be much lower now with the newer generation of preparations, which contain lower doses of estradiol, OC use is still known to contribute to adverse health effects, including hypertension [7,9,10].

Until now, several epidemiological studies have indicated that exposure to OCs may have an effect on blood pressure. OCs has been reported to cause alterations in systolic blood pressure (SBP), diastolic blood pressure (DBP), and the prevalence of hypertension in various study designs with short- and long-term administration [7,11,12]. However, findings from epidemiological studies of the association between OCs and hypertension have been less consistent; according to the population studied, different levels of SBP and DBP have been reported following the administration of OCs. In particular, relatively little information is available about the effect of commonly used OCs containing both estrogen and progestin on blood pressure or the prevalence of hypertension in Korean women.

Alteration of blood pressure profiles may represent an important and potentially etiological component in the pathogenesis of many disorders, including cardiovascular disease, stroke, and renal disease [13]; thus, it is important to examine the association between OC use and hypertension. Thus, the purpose of this study was to investigate this association in Korean women using data from the 2007–2009 Korea National Health and Nutrition Examination Survey (KNHANES), a nationally representative survey conducted in the Republic of Korea.

## Methods

### Study population

This study was based on data from the 2007–2009 KNHANES, provided by the Korea Centers for Disease Control and Prevention. The sample for KNHANES was selected using a stratified, multistage, cluster-sampling design with proportional allocation based on the National Census Registry. Detailed information on survey design and sampling procedures has been reported elsewhere [14]. In total, 600 sampling units were randomly sampled, and 3641 women aged 35–55 years who had no missing response on the questionnaire were included. Of the 3641 participants, 285 participants were excluded because they were taking drugs for hormone replacement therapy (*n* = 272) or were pregnant (*n* = 13). Thus, the final analysis included 3356 subjects.

### Data collection

KNHANES included well-established questions to determine demographic and socioeconomic characteristics of the subjects. These included questions on age, gender, education level, income, physical exercise, smoking habits, and alcohol consumption. Height and weight were measured with the participants wearing light clothing and no shoes. Body mass index (BMI) was then calculated as weight (in kg) divided by the square of height (in m). Then, subjects were categorized as underweight (BMI < 18.5), normal (18.5 ≤ BMI < 23.0), overweight (23.0 ≤ BMI < 25.0), or obese (BMI ≥ 25.0), according to the World Health Organization (WHO) definitions for Asian populations [15]. Information on OC use, including number of months of use, was obtained by a self-administered questionnaire, and blood pressure was measured while subjects were in a sitting position after a 5-min period of comfortable, seated rest. Blood pressure was measured on three occasions with a mercury sphygmomanometer on the right arm, and the second and third measurements were averaged.

The study protocol was approved by the Korean Ministry of Health and Welfare and was conducted in accordance with the Ethical Principles for Medical Research Involving Human Subjects, as defined by the Helsinki Declaration. All study participants provided written informed consent.

### Variable definitions

Hypertensive status was sorted into three categories: normotensive, prehypertensive, and hypertensive. Normotension was defined as not taking an antihypertensive medication and having a SBP of <120 mmHg and DBP of <80 mmHg. Prehypertension was defined as not taking antihypertensive medication and having a SBP of 120–139 mmHg and/or DBP of 80–89 mmHg. Hypertension was defined as SBP ≥140 mmHg and/or DBP ≥90 mmHg or taking medication for hypertension [16]. As a covariate, education level was categorized as less than a high school diploma, high school diploma, and college or higher. Alcohol consumption was assessed by questioning the subjects about their drinking behavior during the month before the interview. The subjects were asked about the average frequency and amount of alcoholic beverage intake. The average amount and number of alcoholic beverages consumed were converted into the amount of pure alcohol (ethanol) consumed per day.

### Statistical analyses

The frequency and percentage or mean and standard error (SE), as appropriate, were calculated for demographic characteristics to describe the sample population according to categories of duration of OC use. Logistic regression models were used to estimate the odds ratio (OR) and 95% confidence intervals (CIs) for prehypertension (vs. normotension) and hypertension status (vs. normotension) among participants who consumed OCs compared with the reference group (those who never used OCs). The presence of a linear trend was evaluated by defining a linear contrast in each of the linear and logistic regression models. All statistical analyses were conducted using SAS software (ver. 9.2; SAS Institute, Cary, NC, USA). Statistical analyses accounted for the survey design, and appropriate procedures in SAS such as surveymeans, surveyfreq, and surveylogistic were used with weighted data.

## Results

This study included 3356 women aged 35–55 years; their demographic characteristics are shown in Table 1. The mean age and mean BMI of the study participants were 44.0 years and 23.5, respectively, and OCs were used by 512 (15.3%) participants. The basic characteristics and outcomes of the study population, sorted by duration of OC use, are presented in Table 2. As duration of OC use increased, participants were more likely to have a low education level (p for trend <0.001). Additionally, duration of OC use was correlated positively with age (p for trend = 0.002), regular exercise (p for trend = 0.041), amount of cigarette smoking (p for trend <0.001), and alcohol consumption (p for trend = 0.044). Average household income was negatively associated with increased OC use (p for trend <0.001).

**Table 1:** Demographic characteristics of participants

**Characteristics**	**N**	**%**
**Age (years)**		
35–39	978	29.1
40–44	841	25.1
45–49	786	23.4
50–55	751	22.4
**BMI**		
<18.5	120	3.6
18.5–22.9	1534	45.7
23–24.9	791	23.6
≥25	911	27.1
**Education**		
< High school	936	27.9
High school	1561	46.5
> High school	859	25.6
**Average household income (US$/month)**		
<1500	916	27.3
1500–2500	1088	32.4
2501–3500	625	18.6
>3500	727	21.7
**Regular exercise**		
Yes	1514	45.1
No	1842	54.9
**Cigarette smoking**		
Never	3075	91.6
Ever	281	8.4
**Alcohol consumption (g/day)**		
0	983	29.3
>0–5.0	1891	56.3
>5.0	482	14.4

**Table 2:** Weighted demographic characteristics by duration of oral contraceptive use

**Characteristics**	**Duration of oral contraceptive use**				**p for trend**
	**Never (n = 2844)**	**<0– < 12 mo (n = 304)**	**12–24 mo (n = 132)**	**>24 mo (n = 76)**	
**Age (years), mean (SE)**	43.9 (0.1)	43.5 (0.3)	44.9 (0.5)	45.5 (0.7)	0.002
**BMI, mean (SE)**	23.4 (0.1)	23.3 (0.2)	23.8 (0.4)	23.7 (0.4)	0.161
**Education (%)**					<0.001
< High school	24.9	35.2	48.5	34.6	
High school	48.5	42.9	37.3	58.1	
> High school	26.6	21.9	14.2	7.3	
**Average household income (US$/month), mean (SE)**	2712.3 (59.5)	2626.2 (103.1)	2278.6 (137.8)	2005.3 (143.2)	<0.001
**Regular exercise (%)**	54.6	53.8	56.9	65.9	0.041
**Smoking (cigarettes/day), mean (SE)**	0.39 (0.05)	0.79 (0.17)	1.11 (0.42)	1.64 (0.49)	<0.001
**Alcohol drinking (g/day), mean (SE)**	3.14 (0.17)	3.41 (0.51)	5.14 (0.82)	4.55 (1.06)	0.044

The overall mean SBP and DBP of the study population were 111.7 and 74.2 mmHg, respectively, and the trends for the weighted mean SBP and DBP were significantly related to increased OC use (Table 3). The weighted mean SBP and DBP were higher among participants using OCs for a longer period of time (p for trend <0.001). Accordingly, a significant dose–response trend existed between duration of OC use and hypertension prevalence (p for trend = 0.001), with 18.1% of the population having hypertension in the group that never used OCs compared with 34.8% in the group that had used OCs for over 24 months. Additionally, the duration of OC use was significantly associated with the prevalence of prehypertension (p for trend = 0.001) and the prevalence of hypertension or prehypertension (p for trend <0.001).

**Table 3:** Weighted distributions of blood pressure-related variables by duration of oral contraceptive use

**Characteristics**	**Overall**	**Duration of oral contraceptive use**				**p for trend**
		**Never (n = 2844)**	**<0– < 12 mo (n = 304)**	**12–24 mo (n = 132)**	**>24 mo (n = 76)**	
Blood pressure (mm Hg), mean (95% CI)*						
Systolic	111.7 (110.7–112.6)	111.4 (110.4–112.4)	111.1 (109.2–112.9)	114.8 (111.0–118.7)	117.2 (113.4–121.0)	<0.001
Diastolic	74.2 (73.6–74.8)	74.0 (73.3–74.6)	74.2 (72.9–75.4)	76.9 (74.5–79.4)	77.8 (75.1–80.5)	<0.001
**Prevalence,%**						
Hypertensive	14.7	14.3	13.9	21.3	22.8	0.006
Prehypertensive	21.3	21.1	21.0	19.2	34.3	0.017
Prehypertensive or Hypertensive	36.0	35.4	34.9	40.5	57.2	<0.001

*Excludes participants who reported being currently treated for hypertension.

**Figure 1:**
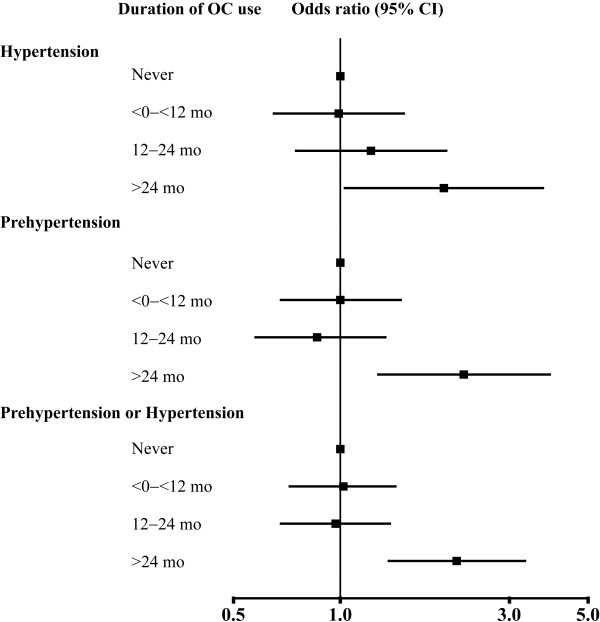
Adjusted odds ratios for hypertension and prehypertension by oral contraceptive use.

Table 4 shows the ORs for the association of hypertension and prehypertension with duration of OC use. The adjusted ORs for hypertension were positively correlated with increased duration of OC use (p for trend = 0.012). Compared with those who never used OCs, the adjusted ORs were 0.99 (95% CI 0.65–1.51) among those who used OCs for 0 to <12 months, 1.22 (95% CI 0.75–1.99) among those used OCs for 12–24 months, and 1.96 (95% CI 1.03–3.73) among those used OCs for >24 months. The trends in adjusted ORs with longer duration of OC use were also positive for prehypertension (p for trend = 0.010) and for hypertension or prehypertension (p for trend = 0.005). Adjusted ORs for prehypertension among the same OC use groups were 1.00 (95% CI 0.68–1.48), 0.86 (95% CI 0.56–1.34), and 2.23 (95% CI 1.28–3.90), respectively, compared with the reference group, and adjusted ORs for hypertension or prehypertension were 1.02 (95% CI 0.72–1.43), 0.97 (95% CI 0.68–1.38), and 2.13 (95% CI 1.37–3.32), respectively (Figure 1).

**Table 4:** **Odds ratios and 95**% **confidence intervals for hypertension and prehypertension by duration of oral contraceptive use**

	**Duration of oral contraceptive use**				**p for linear trend**
	**Never (n = 2844)**	**<0– < 12 mo (n = 304)**	**12–24 mo (n = 132)**	**>24 mo (n = 76)**	
**Hypertension**					
Crude	1.00 (reference)	0.97 (0.65–1.43)	1.63 (1.05–2.52)	2.42 (1.43–4.09)	<0.001
Adjusted*	1.00 (reference)	0.99 (0.65–1.51)	1.22 (0.75–1.99)	1.96 (1.03–3.73)	0.012
**Prehypertension**					
Crude	1.00 (reference)	0.99 (0.68–1.43)	0.99 (0.64–1.53)	2.46 (1.44–4.19)	0.001
Adjusted*	1.00 (reference)	1.00 (0.68–1.48)	0.86 (0.56–1.34)	2.23 (1.28–3.90)	0.010
**Prehypertension or Hypertension**					
Crude	1.00 (reference)	0.98 (0.71–1.34)	1.24 (0.90–1.73)	2.44 (1.63–3.66)	<0.001
Adjusted*	1.00 (reference)	1.02 (0.72–1.43)	0.97 (0.68–1.38)	2.13 (1.37–3.32)	0.005

*Adjusted for age, BMI, education, income, exercise, cigarette smoking, and alcohol consumption.

## Discussion

OCs containing estrogen and progestin constitute a widely used means of contraception. Currently used third-generation OCs were designed to minimize adverse effects, including unfavorable effects on the cardiovascular system [17]. Since their introduction, a number of studies have confirmed that their effects on blood pressure are indeed less than those observed with first- and second-generation OCs [18,19]. However, controversy persists about the possible risks of OC use, including cardiovascular disease, partially through the promotion of adverse changes in blood pressure [20].

In the present nationwide cross-sectional study among Korean women aged 35–55 years, the duration of OC use was positively associated with SBP and DBP levels. SBP and DBP among those who had used OCs >24 months were higher than among those who had never used them by 5.8 and 3.6 mmHg, respectively. Similar to the results of this study, most studies have reported a steady increase in SBP and DBP among users of third-generation OCs containing estrogen and progestin. However, the magnitude of the increase varies among populations and also with the duration of OC use. Case–control studies using 24-h ambulatory BP monitoring indicated a higher SBP and DBP in OC users than in non-users of about 7.6–8.6/1.8–5.8 mmHg [21,22]. Also, a prospective study of approximately 18 000 US women reported an increase in SBP and DBP by 5–6 mmHg and 1–2 mmHg, respectively, in white women [23,24]. Another study conducted by WHO reported that women using OCs showed increased SBP and DBP, by 3.6–5.0 mmHg and 1.9–2.7 mmHg, respectively, compared with those using an intrauterine device [25]. A similar finding has been reported in German women; OC users had, on average, significantly higher levels of SBP and DBP than non-users by ~3.6 and ~1.4 mmHg, respectively [26]. However, SBP and DBP among current OC users were higher than those among never users by only 0.7/0.4 mmHg in a prospective cohort study in the United States [12]; furthermore, SBP increased by only 2 mmHg and no change was found in DBP in British women between the ages of 18 and 30 years [27]. A possible explanation for these differing effects of OCs on blood pressure among studies may be differences in OC formulations and treatment regimens as well as the age and race/ethnicity factors of participants. Thus, it is important to examine the effect of OCs on blood pressure in a Korean population exposed to third-generation OCs for different periods of time.

In this study, hypertension, prehypertension, or prehypertension + hypertension were about twofold more common in women taking OCs for more than 2 years than in women not taking OCs. However, no significant increase was observed among women who had used OC use for less than 2 years in the odds of hypertension, prehypertension, or prehypertension + hypertension. Although the cumulative time of OC use shown to produce an increased odds of hypertension varies by study, long-term effects of OC use on hypertension have been confirmed in many studies [7,11,12]. Prehypertension is a precursor of clinical hypertension and is associated with an increased risk of cardiovascular disease [28,29]. Given that the prehypertension, as well as hypertension, is an important risk factor for cardiovascular morbidity and mortality [5,30], data from this study suggest that taking OCs for more than 2 years may be a risk factor in the development of cardiovascular disease, leading to an increased risk of premature death in Korean women.

Although the detailed biological mechanism of the hypertensive risk due to exogenous estrogen and/or progestin remains to be determined, estrogen and progesterone are known to regulate many intracellular signal transduction and cellular functions [17,31]. Estrogens acting on estrogen receptors α and β are recognized as important regulators of intracellular signaling cascades [32]. Many studies have demonstrated that estrogens regulate vascular tone, mediated by nitric oxide, prostacyclin, angiotensin, and the sympathetic nervous system [33-35]. Moreover, the type and dose of progestin in OCs can influence blood pressure [36,37]. For example, it has been reported that progesterone-induced increases in aminopeptidase P may contribute to the development of OC-induced hypertension in susceptible women [38].

The present study has several limitations. As a result of the cross-sectional design, the results only demonstrated associations and could not be used to determine causality. Additionally, self-reporting of drinking and smoking patterns and of OC use may lead to misclassification and measurement error [39]. Although this study included adjustment for many potential confounding factors, the results may have been confounded by unmeasured covariates, such as type of OC formulation and pre-existing diseases. Furthermore, although genetic and enzymatic factors are important in the metabolism of OCs and in the homeostatic regulation of blood pressure, the results of this study could not determine how the association of OCs with blood pressure may be affected by these factors. Despite these limitations, this study had several major strengths. To our knowledge, this is the first reported study to assess the association between duration of OC use and hypertension or prehypertension in Korean women using nationally representative data. Another strength is that this study analyzed not only hypertension status but also prehypertension following OC use. Future studies investigating the mechanisms underlying the relationship between OCs and blood pressure and any racial/ethnic differences among populations are required to confirm and extend the results of this study.

## Conclusion

In this population-based study of Korean women aged 35–55 years, long-term use of OCs was associated with increased SBP and DBP. Additionally, odds of hypertension or prehypertension was significantly increased among women using OCs for more than 2 years compared with non-users. Although controlled studies to investigate causal relationships between OC use and blood pressure are needed, the present results suggest significant associations between long-term use of OCs and increased hypertension.

## Competing interests

All authors declare that they have no competing of interests.

## Authors’ contributions

Dr HP contributed to data analysis and interpretation, statistical analysis, and drafting the manuscript. Dr KK contributed to design of the study, critical revision of the manuscript, and supervision of the study. Both authors have read and approved the final manuscript.

## Pre-publication history

The pre-publication history for this paper can be accessed here:

http://www.biomedcentral.com/1472-6874/13/39/prepub
